# Sandwich Structures for Energy Absorption Applications: A Review

**DOI:** 10.3390/ma14164731

**Published:** 2021-08-22

**Authors:** Faris Tarlochan

**Affiliations:** Department of Mechanical and Industrial Engineering, Qatar University, Doha 2713, Qatar; faris.tarlochan@qu.edu.qa

**Keywords:** sandwich structure, foam, honeycomb, auxetic structure, architected core, crashworthiness, blast, impact

## Abstract

It is crucial that proper engineering structures are designed as energy absorbers for high dynamic loading situations, such as accidents, blasts, or impacts. The role of such structures is to absorb the high kinetic energy as strain energy through irreversible deformation of the structure. Many types of energy absorbers were designed for different dynamic high strain rate applications. One of these structures are sandwich structures. The aim of this review paper is to provide a general review on the type of sandwich structures that have been designed as energy absorbers and their performance in crashworthiness and blast related applications. The focus is on the type of core structures being used, namely foam and architected cores. It was found from the review that sandwich structures are viable candidates for such applications not only because of their light weight, but also due to the high-energy absorption capabilities. The work presented in this review paper shows that the data from the literature on this topic are vast and do not converge to any particular sandwich structure design. This presents the potential future research direction in designing sandwich structures, which have wider application at different scales.

## 1. Introduction

**Figure 1:**
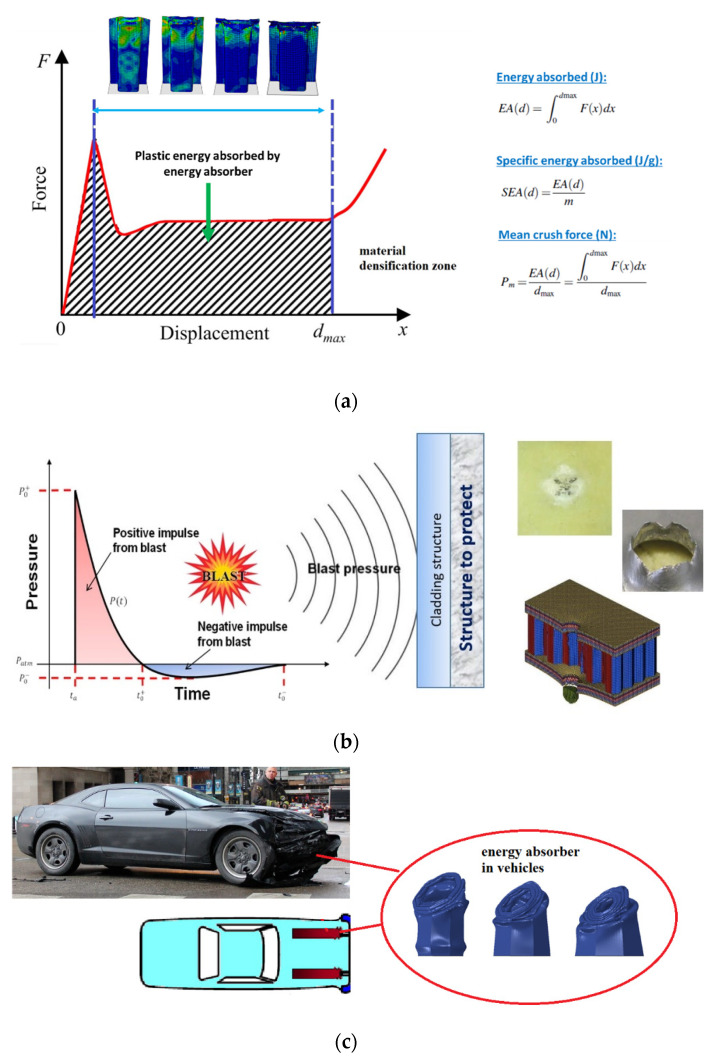
(**a**) General energy absorption through deformation of energy absorbers, (**b**) energy absorbers used as cladding structures for blast resistant applications and. (**c**) energy absorbers used in vehicle safety. [partial image source: “Aftermath of Car Crash on Randolph at Michigan, 21 January 2015” by danxoneil is licensed under CC BY 2.0].

Engineering structures are designed and developed for many applications, such as load bearing (fatigue or static), high-pressure containment, safety, energy absorption, etc. These structures come in various design configurations, geometries, materials, loading conditions, physical constraints, etc., each one unique to the type of application. The focus of this review paper is to look at engineering structures designed primarily for energy absorption applications, particularly sandwich structures, due to the current engineering requirements for lightweight structures [1]. Energy absorbing structures are designed primarily to absorb energy during a dynamic event, such as a high strain rate event like an impact (due to collisions) or blast [2,3,4,5,6,7,8,9]. Other areas such as the cargo/goods packaging sectors also require structures that are able to absorb “impact” energy during handling and transportation. This paper, however, is focusing just on structures used for absorbing energy during high strain rate events, in particular crashworthiness and blast related. The motivation of this paper is to consolidate findings by researchers in the area of using sandwich structures as energy absorbers in high dynamic events and to identify future research directions to enhance/strengthen this area of research (applications to crashworthiness and blast related). Crashworthiness is defined as the extent to which a vehicle is able to protect its occupants in the event of a collision or accident. In the area of blast applications, sacrificial or cladding structures are designed to absorb the energy in the event of a blast to protect the primary structure due to the blast wave and perforations due to blast projectiles like shrapnel. Both applications, crashworthiness and blast resistant, require structures that are able to absorb energy to protect people or cargo from serious damage or injuries. In such applications, energy absorbers are designed to absorb the change in kinetic energy (during an accident or blast) into strain energy that is then used to deform the energy absorber. Some of the parameters used for analysis are energy absorption *(EA)*, specific energy absorption *(SEA),* and mean crush force *(P_m_)*. The literature documents various designs of energy absorbers based on different materials, loadings, and geometries, similar to those reported in [2,3,4,5,6,7,8,9]. Graphical details of such applications are depicted in Figure 1. In Figure 1a, *d_max_* represents the maximum crushed distance before densification starts, or where the deformation (crushing) stops, whereas *m* represents the crushed mass.

With reference to Figure 1b, when a detonation takes place, a huge amount of energy is released in an unconfined medium, giving rise to blast wave expanding in all directions. At some fixed point away from the point of blast, there is a rapid peak in pressure followed by a rapid decay. The time the shock arrives here is called time or arrival of shock and is denoted as *t_a_*. Sometimes, the static pressure will fall below atmospheric but will eventually equilibrate (negative pulse). However, this depends on the conditions of blast and the distance from the blast location. The rise in pressure is due to the shock wave moving forward from the point of blast. Positive pulse duration is the time when the pressure is positive and this helps to define the positive impulse, which is the area under the curve. For blast mitigation applications, this positive impulse is used for designing the energy absorbers.

**Figure 2:**
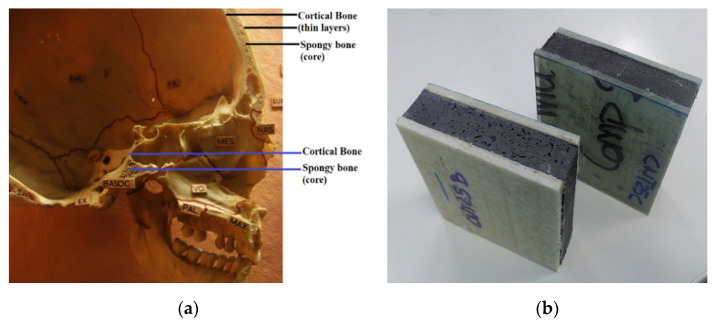
(**a**) Human skull a nature’s design of sandwich structure for energy absorption. (Source of image: “Human Skull” by Quasimondo is licensed under CC BY-NC 2.0) (**b**) Example of sandwich panel (Source of image: “NANOCORE sandwich panel with MWCTs” by JavierACCIONA is licensed under CC BY-NC 3.0).

Among the common engineering structures used for energy absorption application are sandwich structures. A typical sandwich structure consists of two face sheets separated by a lightweight thick core structure such as foams or honeycombs. Lightweight sandwich structures are used extensively in aerospace, marine, and automotive industries due to the high flexural stiffness-to-weight ratio and excellent energy absorption capability [10]. The idea of sandwich structures for energy absorption applications is actually not a new concept. Rather, they are inspired by nature, e.g., the human skull that comprises two layers of dense cortical bone separated by a spongy bone (core–cancellous bone) to protect the brain from small impacts (Figure 2) [11]. Having this in mind, and the requirement for lightweight materials, the paper will discuss works related to sandwich structures used for potential energy absorption related applications. Since the principal energy absorption is due to the deformation of the sandwich core structure [12], the paper will be organized based on two core structure configurations: (a) cellular foam cores and (b) architected cores.

## 2. Sandwich Structures for Energy Absorption

This section will contain the literature review on sandwich structures used as energy absorbers. In sandwich structures, the type of high strain rate application will determine the way a sandwich structure is designed. In blast wave/crashworthiness applications, if the sandwich panel is designed to be compressed flatwise, the core plays a crucial role in the energy absorption, compared to the face sheets. However, if the panels are designed for edgewise compression, both the face sheets and core play a role in the energy absorption. For perforation applications due to blast, there is penetration of the structure due to some projectiles. Here, again, the combination of the face sheets and core play a vital role in improving impact resistance. In all the above applications, the failure mechanism of the sandwich structure/panel is a crucial element in the energy absorption capabilities of a structure. Local buckling of core cell wall structures (foam or architecture cores), core shearing, indentation, face sheet yielding, wrinkling, and interlaminar failure of face sheets are some of the failure mechanisms observed in during these applications. The review will be broken down into two sections based on the type of core used: (1) foam cores and (2) architected cores.

### 2.1. Cellular Foam Core Structure

Foams are lightweight structures that can absorb a good amount of energy when stressed to their plateau region (plastic deformation) in the stress–strain diagram, as described by Gibson and Ashby [13]. A typical constant plateau is about 60–70% of the total strain value [13]. The beauty of foams is that their properties are heavily dependent on their density, hence allowing designers the capability to develop foams unique for its applications [14,15]. The foams are fabricated as open cell or closed cell structures, where the former leads to a lighter structure because the cells structures in the foam are not completely encapsulated. In the literature, significant results have been presented concerning the ability of metallic and polymeric foams as standalone structures for energy absorption applications. This review paper will only focus on sandwich structures that utilizes these foams as structural cores.

#### 2.1.1. Polymeric Foam Core

Some sandwich structure designs utilize polymeric foams as the core material due to the cost and ease of fabrication compared to metallic foams. Table 1 details a summary of some of the related applications of such sandwich structures. The typical applications are for low velocity impact (perforation), shock wave, and crashworthiness applications. For sandwich structure face sheets, typical materials used are either metals or fiber composites. For the core, various polymers have been used, such as polyvinyl chloride (PVC), polyethylenimine (PEI), polystyrene (PS), polyurethane (PU), polymethacrylimide (PMI), and styrene acrylonitrile (SAN), to name a few. Some of the key findings are as follows:

- Graded polymeric cores (varying densities) are better than uniform density cores
- Different polymer cores perform differently
- The type of boundary conditions used affected the blast mitigation strategy
- Failure mechanisms of the composite face sheets and core play a vital role in the energy absorption capability
- Parametric design is crucial to optimize the sandwich structure for energy absorption based on application type
- The type of blast (near vs. far field) creates different responses on the sandwich panel
- Sandwich structures used for crashworthiness related applications/testing conditions demonstrated progressive crushing

#### 2.1.2. Metallic Foam Core

For higher energy absorption performance, sandwich structures designed with metallic foams are used. Table 2 details a summary of some of the related applications of such sandwich structures. The typical applications reported in the literature are for low velocity impact (perforation), shock wave, and crashworthiness applications. For sandwich structure face sheets, typical materials used are either metals (stainless steel/aluminum) or fiber composites. For the core, the most common foam material used was aluminum. Some of the key findings are as follows:

- Graded metallic cores are better than uniform density cores.
- Failure mechanisms of the face sheets and core play a vital role in the energy absorption capability.
- Parametric design is crucial to optimize the sandwich structure for energy absorption.
- For perforation applications, it was found that the sandwich panels performed poorly compared to the monolithic aluminium panel.
- Sandwich structures used for crashworthiness related applications/testing conditions demonstrated progressive crushing.
- The blast resistance of the sandwich panels comprised of the composite face sheets outperformed the metallic counterparts.
- Strain rate of the foam core is important in defining the crushing behavior which is linked to the energy absorption capabilities.

### 2.2. Architected Core Structure

Foams, especially the metal foams, have some challenges. One of the common challenges is the non-uniformity of the cell structure for the foam due to the existing fabrication process (foaming of melts/powder). This results in a lack of efficiency in batch or mass production, especially when producing near-net products or tailoring for customized applications. This is true when one wants to fabricate functionally graded metal foams, for example. Due to enhancements in manufacturing technology such as 3D printing, new cores can be design and developed to fulfill certain functional requirements (architected core). This review will cover such cores as: (a) honeycomb cores; (b) truss/lattice structure cores; (c) origami/fold-cores; (d) auxetic core, and (f) tubular cores.

#### 2.2.1. Honeycomb Structure

**Figure 3:**
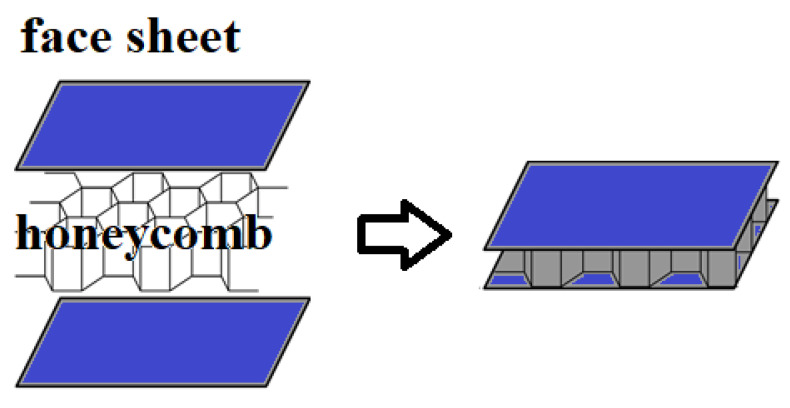
Honeycomb sandwich panel.

Honeycomb sandwich structures (Figure 3) are one of the earliest architected core sandwich structures used for dynamic loading events. Table 3 details a summary of some of the related applications of such sandwich structures. The typical applications reported in the literature are for low velocity impact (perforation), shock wave, and crashworthiness applications. For sandwich structure face sheets, typical materials used are either metals (stainless steel/aluminum) or fiber composites. For the core, the most common honeycomb material used was aluminum followed by polymeric and paper (Nomex). Some of the key findings are as follows:

- Graded honeycomb cores are better than uniform density cores.
- Honeycomb geometry plays a vital role in the energy absorption capability.
- Failure mechanisms of the face sheets and core play a vital role in the energy absorption capability.
- Parametric design is crucial to optimize the sandwich structure for energy absorption.
- For perforation applications, it was found that most of the energy absorption is due to the face sheet of the panels.
- Sandwich structures used for crashworthiness related applications/testing conditions demonstrated progressive crushing, especially with honeycomb that is filled with foam.
- Strain rate of the honeycomb is important in defining the crushing behavior, which is linked to the energy absorption capabilities.

#### 2.2.2. Truss/Lattice Like Structures as Core

**Figure 4:**
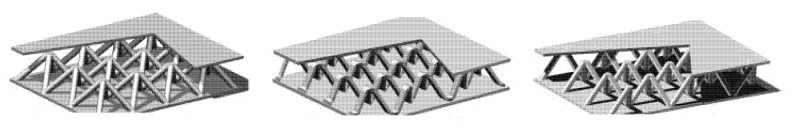
Lattice core sandwich panel. (image source: “Creative Commons Multifunctional sandwich panel with metallic lattice cores” by Zhang et al. https://doi.org/10.3390/en10070906 (accessed on 18 August 2021), used under CC BY 4.0, modified from original).

Sandwich structures with a truss/lattice structure (Figure 4) were adopted as the core is a new type of architected core sandwich structure used for dynamic loading events. Table 4 details a summary of some of the related applications of such sandwich structures. The typical applications reported in the literature are for low velocity impact (perforation), shock wave, and crashworthiness applications. For sandwich structure face sheets, typical materials used are either metals (stainless steel/aluminum) or fiber composites. For the core structure, the most common material used was metal followed by polymer. Some of the key findings are as follows:

- Higher aerial density of the truss like structure enhances the energy absorption capabilities.
- The truss/lattice core with foam filling enhances the energy absorption and impact resistance capabilities.
- The empty lattice core does not support perforation related applications.
- The type of lattice/truss structure geometry design affects the energy absorption capabilities.
- Failure mechanisms of the face sheets and core play a vital role in the energy absorption capability.

#### 2.2.3. Origami/Foldcore Structures

**Figure 5:**
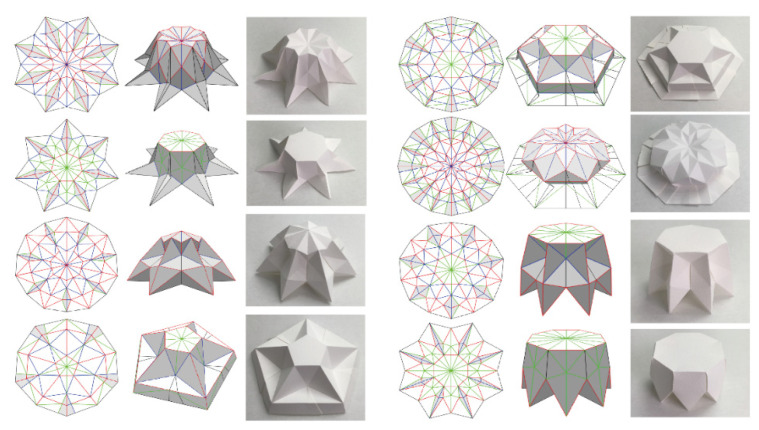
Example of origami patterns that can be used to construct the core of a sandwich panel. (image source: “Creative Commons Multifunctional resulting origami pieces with 3D flaps” by Zhao et al. https://doi.org/10.3390/sym10100469 (accessed on 18 August 2021), used under CC BY 4.0).

Sandwich structures with origami type structure were adopted as the core is a new type of architected core sandwich structure used for dynamic loading events. Figure 5 depicts some example of origami patterns that can be used to construct the core of a sandwich panel or beam. Table 5 details a summary of some of the related applications of such sandwich structures. The typical applications reported in the literature are for low velocity impact (perforation), shock wave, and crashworthiness applications. For sandwich structure face sheets, typical materials used are either metals (stainless steel/aluminum) or fiber composites. For the core structure, the most used material was metal followed by polymer. Some of the key findings are as follows:

- Perforation energy is highly related to the origami wall thickness.
- The origami structure provides multiple hinges for plastic deformation, which enhances energy absorption capabilities.
- Geometrical parameters of the origami are crucial to the development and optimal design for energy absorption.
- The origami sandwich structure was found to be better than the honeycomb sandwich structure.
- The origami structure can be optimized and tailored easily for various dynamic related events.

#### 2.2.4. Auxetic/Meta-Structured Core Structures

**Figure 6:**
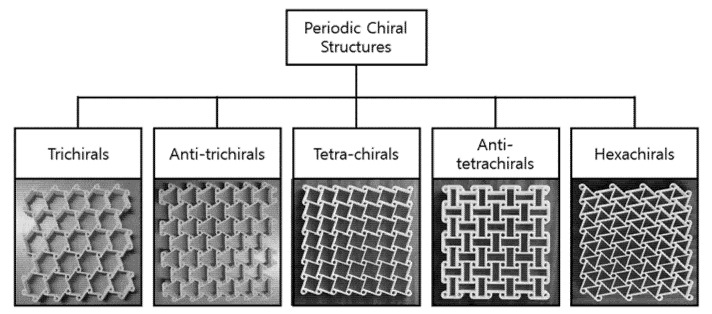
Example of auxetic structure that can be used to construct the core of a sandwich panel. (image source: “Creative Commons Multifunctional classification and representative structures of periodic chiral structures” by Kelkar et al. https://doi.org/10.3390/s20113132 (accessed on 18 August 2021), used under CC BY 4.0).

Auxetic core structures (Figure 6) are structures with a negative Poisson’s ratio. As a new class of material/structure, it has been studied recently. Such auxetic structures, when pulled, become thicker in the direction perpendicular to the force. Further, 3D printing can be used here to fabricate such structures easily where the face sheet is printed on the core, overcoming the limitation of delamination/debonding of the face sheet from the core. Sandwich structures with such a core structure represent a new type of architected core sandwich structure used for dynamic loading events. Table 6 details a summary of some of the related applications of such sandwich structures. The typical applications reported in the literature are for low velocity impact (perforation), shock wave, and crashworthiness applications. For sandwich structure face sheets, typical materials used are either metals (stainless steel/aluminum) or fiber composites. For the core structure, the most common material used was metal followed by polymer. Some of the key findings are as follows:

- The core design geometrical parameters have significant effects on the failure mechanism and energy absorption of the auxetic structures.
- The geometry parameters, such as thicknesses and core density, affect the ballistic resistance performance.
- It was also found that auxetic honeycomb, as a core in sandwich panels, provides good ballistic protection.
- The auxetic sandwich panel has good energy absorption capabilities.

#### 2.2.5. Tubular Core-Like Structures

**Figure 7:**
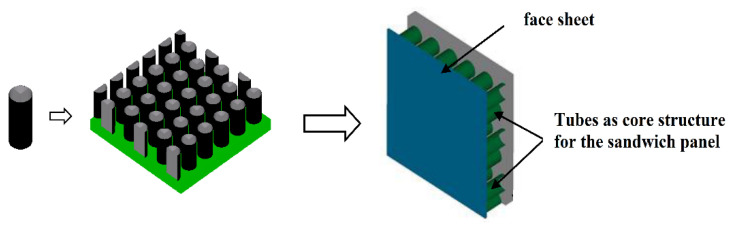
Example of tubular sandwich structure.

For this type of sandwich structure, the core structure is comprised of tubes (Figure 7). Table 7 details a summary of some of the related applications of such sandwich structures. The typical applications reported in the literature are for low velocity impact (perforation), shock wave, and crashworthiness applications. For sandwich structure face sheets, typical materials used are either metals (stainless steel/aluminum) or fiber composites. For the core structure, the most common material used was metal followed by polymer. Some of the key findings are as follows:

- Such a core design provides good blast resistance and crashworthiness, although less perforation related.
- The tube arrangement between the face sheets is crucial because it affects the plastic hinge formation.
- Tubes filled with foams have good energy absorption capabilities.

#### 2.2.6. Corrugated Core-Like Structures

Corrugated cores in sandwich panels are easy to construct and have been shown to perform well under compression testing. Table 8 details a summary of some of the related applications of such sandwich structures. The typical applications reported in the literature are for low velocity impact (perforation), shock wave, and crashworthiness applications. For sandwich structure face sheets, typical materials used are either metals (stainless steel/aluminum) or fiber composites. For the core structure, the most common material used was metal followed by polymer. Some of the key findings are as follows:

- Corrugation buckling and fracture are the main failure mode.
- The corrugated core can be viewed as a subset of the origami core design. As such, the failure mechanism characteristics are similar to those of the origami cores.

## 3. Discussion and Research Direction

From the literature review presented above, it is obvious that sandwich structures are good candidates for energy absorption applications. The traditional panels made from foam and honeycomb cores are being challenged against newly architected cores. In principle, the failure mechanism of such sandwich structures play a crucial role in determining the energy absorption capabilities. As such, the usage of sandwich structures for energy absorption applications requires extensive design parametric studies to optimize the panel for a certain application, as shown from the literature review.

The work published thus far is scattered in terms of potential real-life application. It is not possible to support any sandwich structure design as the ultimate energy absorber design because a lot of material configurations, structure size, loading conditions, and structure geometry conditions were tested and investigated. It is an uphill task to normalize the findings of such works. Some of these have not been tested at different scales of size or different loading conditions (example strain rate sensitivity). In view of this, the future direction of sandwich structures for energy absorption should be as follows:

- The development of a more comprehensive experimental scheme that will allow for the performance evaluation of a particular sandwich structure design based on parametric study to understand the structure response from different scales of size and at different high strain dynamic events.
  ∘ This should be supported by numerical simulation to reduce cost and to expedite the understanding of the structure’s response.
  ∘ The findings should be linked to the failure mechanism and used to develop a design map that allows to better understand the effect of selecting different parametric variables on the desired performance (to identify design rules).
  ∘ Similar approach should be conducted on different core design and material (including face sheets).
- Based on the extensive experimental supported by a numerical simulation scheme (as discussed previously), there will be a need to use artificial intelligence/data mining with topology optimization to design sandwich structure(s) for a particular application. Some of the recent works in using artificial intelligence in design can be found in the following works [117,118,119,120,121].
- Studies should also be performed to assess the effect of small damages on the crashworthiness or blast performance. Residual/minor indentation due to either manufacturing defects or human handling of the structure can affect the overall performance. Such work is yet to be reported within the scope of sandwich structures as energy absorbers.
- Issues related to manufacturing: The ease of fabrication, ease of maintenance, impact on environment, sustainability, scaling up (mass production), and life-cycle cost analysis are not well discussed. There is a need to map the overall sandwich structure performance with such indicators. This will help designers select the most appropriate sandwich structure design.

## 4. Conclusions

This review paper addresses the usage of sandwich structures for energy absorption applications. It was found that such sandwich structures are good candidates to be designed as energy absorbers. Depending on the type of loading conditions, it was observed that the failure mechanism of such structures is highly dependent on the core geometry and design variables, such as core thickness, cell thickness, face sheet thickness, type of material, etc. The review shows that the work in this area is vast and does not converge to any particular structure design. There is good potential in using sandwich structures, but these structures need to be designed in a more intelligent way to fully realize their potential. As such, the future direction of designing such structures is through the usage of artificial intelligence/data mining coupled with topology optimization.

## Figures and Tables

**Table 1:** Sandwich structure with polymeric foam core.

Sandwich Construction	Loading/ Application	Summary of Findings	Reference
PVC, cross-linked PVC and PEI foams were bonded together to produce a three layer core bonded with carbon fibre face sheets	Low velocity Impact (perforation-experiment)	Improved perforation resistance was achieved when the highly dense layer was attached to the top skin.	[16]
E-Glass fiber composite face sheets with graded styrene foam cores different densities	Shock wave loading (blast-experiment)	The low/middle/high density foam configuration had better energy absorption capabilities compared to other configurations of the foam densities.	[17]
Sandwich panel consisting of E-glass fiber composite face sheets and H100 PVC foam core	Blast loading (analytical solution)	Two phases of deformation were identified: (a) core crushing during through-thickness wave propagation and (b) global panel bending/shear during transverse shear wave propagation	[18]
Sandwich composites made of E-glass fiber composite face sheets and graded Corecell™ A-series foam	Shock wave loading (blast-experiment)	Designing the foam cores to be graded monotonically, helps improve blast resistant performance. At higher temperatures, the failure mechanism of the cores differed to the lower temperatures.	[19,20]
Sandwich material with a soft layer (PU) in between woven composite skins (EVE)	Shock wave loading (blast-experiment)	Sandwich panels had better blast resistance performance than composite plates.	[21]
Sandwich panels comprising E-glass fibre reinforced vinyl ester face sheets and closed cell PVC foam cores	Blast loading (blast-experiment)	Face-sheet delamination and fiber fracture along with core compression were significant energy absorption modes. For low core densities, the face sheets absorbed more energy due to the blast.	[22]
Glass fiber reinforced sandwich panels with PVC, PMI and SAN foam cores	Air blast testing (blast-experiment)	All three type of polymer cores are effective in improving blast resistant where the SAN core was the most blast tolerant. It was also found that by grading the core densities, a smoother back face-sheet deflection profile was achievable.	[23]
(1) E-glass fabric face sheets with balsa wood core, (2) E-glass fabric face sheets with PVC foam core, (2) E-glass fabric face sheets with balsa wood core, (2)	Large air blast testing (blast-experiment)	Failure mode maps were developed to provide insights on how panel failure depends on the key variables during a blast event	[24]
E-glass face sheets and a Styrene Acrylo-Nitrile (SAN) foam core	(a) Primary: a high velocity projectile or a low velocity drop weigh followed by (b) Secondary: blast loading (impact/blast—experiment)	The damage due to low velocity drop weights had the greatest detrimental effect on the blast performance of the sandwich composites. This is due to the initial failure of the panels, which involves debonding of the face sheets and shear cracking of the core.	[25]
3-D woven 3WEAVE^®^ E-glass fiber composites skin preforms integrally stitched to polyisocyanurate TRYMERTM 200L foam core	Shock wave loading (blast-experiment)	Through-thickness stitching with foam core increases the shock wave resistance and damage tolerance. It was also found that the prominent damage mechanism differs for unstitched to stitched sandwiches, where increasing stitching density changes the damage mechanism.	[26]
E-glass quadriaxial skins with SAN foam core (Large panels)	Air Blast loading (blast experiment)	The type of boundary conditions (type and location) plays and important role in blast mitigation applications.	[27,28]
Analytical model of metallic sandwich with soft and hard cores	Water Blast loading (Blast-analytical and simulation)	Sandwich plates with stiff cores imparted higher blast impulses compared to those with softer cores and equivalent areal mass	[29]
Sandwich panels with Divinycell H-100 PVC foam and glass fiber epoxy face sheets	Water Blast loading (Shock tube)(blast experiment)	Sandwich panels with face sheet-thickness-to-core-thickness ratios between 0.15 and 0.4 provided the best blast resistance design	[30]
Sandwich composites made of E-Glass Vi-nyl-Ester (EVE) face sheets and graded Core-cell™ A-series foam	Air blast loading (blast experiment)	Due to the buckling of the face sheet (in plane compressive loading), the blast resistance efficiency reduced, indicating to some extend the face sheets play an important role in blast resistant designs.	[31]
Foam-core, curved composite sandwich pane	Air Blast loading (Blast-analytical and simulation)	Blast resistance increases when the sandwich cores are allowed to undergo plastic deformation. Besides this, it was found that dense foam cores did not increase the blast resistance but allowed face sheets to fracture while the core remained elastic.	[32]
Double-curvature, sandwich shallow shell with PVC foam (face sheet E-Glass/Vinyl Ester Woven Roving)	Air Blast loading (Blast-analytical model)	(1) Blast resistance increases as the panel curvature ratio decreases because shells, (2) blast resistance of isotropic core is higher than transversely isotropic core as the shell radius of curvature decreased.	[33]
Sandwich panel: nano-scale core-shell rubber (CSR) toughened E-glass Vinyl-Ester face-sheets and Corecell A500	High pressure shock (blast—experiment)	CSR particles helps in dispersing the initial shock wave loading, thus improving the overall blast resistance of the structure	[34]
Sandwich panels face sheets made up from glass and carbon fibers, with PVC foam core.	Air Blast loading (blast—experiment)	Under large-scale blast loading where the load is almost uniform across the panel, the type of face sheet material does not influence much on the panel deformation.	[35]
Sandwich structure consists of glass fiber composites as face sheets, and core is rigid polyurethane	Gas gun projectile impact (perforation- experimental)	The density of the core material plays an important role in ballistic performance requirements. Neither too rigid nor too soft cores are desirable. This is related to the foam’s cell wall thickness and strut.	[36]
Sandwich structures face sheets made from glass fiber, core is polystyrene foam	Compression Test (Crashworthiness—experimental)	Sandwich structure collapsed in a progressive manner exhibiting high-energy absorption capabilities.	[37]
Sandwich structures face sheets made from glass and carbon fiber composites, core is polystyrene foam	Compression Test (Crashworthiness—experimental)	Four failure modes were observed with the primary mode of failure was progressive crushing due to the foam as core structure. The optimized design had a very good specific energy absorption capability.	[38]

**Table 2:** Sandwich structure with metallic foam core.

Sandwich Construction	Loading/ Application	Summary of Findings	Reference
Spherical shell sandwich made from aluminum metallic foam cored with varying densities with outer and inner face sheet made from aluminum	Inner blast loading—from inner center of the structure (blast-simulation)	The arrangement of core foam density play an important role in optimizing the blast resistant response	[39]
Square sandwich panels made up from aluminum alloy face-sheets and a layered gradient aluminium foam core	Air-blast loading (blast-simulation)	Blast resistant efficiency depends on how the layered gradient foam cores are arranged. Besides this thickness of the face sheet has little influence on the blast resistance.	[40]
Three types of face sheet materials (aluminum alloy Al6061, glass fiber and carbon fiber reinforced plastic, with aluminum foam core	Sequential low-velocity off-panel pre-impact and/or in-panel post-compression tests (Crashworthiness—experiment)	The type of loading conditions affects different material face sheets, but overall the fiber-reinforced plastics performed better.	[41]
Sandwich panels made from closed-cell aluminium foam cores and aluminium face sheets	Low velocity Impact (perforation-simulation)	For perforation application, it was found that the sandwich panels performed poorly compared to the monolithic aluminium panel.	[42]
Sandwich panel consists of aluminum foam core with steel as face sheets	Air-blast loading (blast—simulation)	Specific energy absorption increases with the increase of foam thickness and the sandwich panel can reduce peak acceleration by 50% compared to steel plates.	[43]
Sandwich panel core is from closed-cell aluminum foam. The face sheets consists of three different materials (304 stainless steel, 5182 aluminum alloy and carbon fiber composite).	Pendulum Blast test (blast- experiment)	The blast resistant of the sandwich panels comprised of the composite face sheets outperformed the metallic counterparts	[44]
Aluminum/foam/CFRP hybrid sandwich tubes	Compression test (Crashworthiness—experiment and simulation)	Better crashworthiness characteristics to cost of these hybrid structures were identified.	[45]
Steel plates as face sheets with aluminum foam as core	Compression test (Crashworthiness–simulation)	The usage of sandwich structure increased the energy absorption capability for the selected application.	[46]
Sandwich panels face sheets made composite and aluminum whereas the core was aluminum foam	Low velocity impact test (perforation- experiment)	Sandwich panels with aluminium face sheets showed higher SEA than composite face sheets. It also performed better in other design constraints such as cost, impact to environment, etc.	[47]
Sandwich panel core is from closed-cell aluminum foam. The face sheets consists of three different materials (304 stainless steel, 5182 aluminum alloy and carbon fiber composite).	Ballistic Pendulum (Blast—experiment and simulation)	The sandwich structure with descending gradient density of the foam core provided the highest blast resistance. Even and uneven face sheet thickness influence the blast resistant performance, depending on the intensity of the blast	[48]
Double wall tubular structure filled with metallic aluminum foam.	Compression test (Crashworthiness—experiment and simulation)	(1) strain rate of the foam core is important in defining the crushing behavior which is linked to the energy absorption capabilities. (2) Interaction between the foam and the tube wall enhance multiple propagating folds, which enhance crashworthiness performance.	[49]
Functionally graded close-celled aluminum foam cores with stainless steel face sheets	Air blast loading (Blast—simulation)	Sandwich panels with graded foam core possess smaller central transverse deflection and superior blast resistance.	[50]

**Table 3:** Sandwich structure with honeycomb core.

Sandwich Construction	Loading/ Application	Summary of Findings	Reference
Aluminium face sheets with triple layered graded honeycomb cores	Blast loading (blast—experimental and finite element)	Effects of geometric configuration on type of deformation modes and blast resistance were studied. Core arrangement affected the energy absorption and blast resistance capabilities. Core with higher density arranged closer proximally performed better.	[51]
Steel face sheet with aluminium honeycomb core	Blast loading—uniform and localized (Blast—experiment)	Failure performance or blast resistant was better for uniform loading than under localized loading. It was also found that the load transfer to the back face sheet depends on the load intensity, core thickness and flexibility of the sandwich structure.	[52,53]
Sandwich panel steel face sheets with unbounded aluminium foam or hexagonal honeycomb cores	Air Blast loading (Blast—experimental)	Face sheet thickness has a significant effect on the blast resistance performance for both the foam and honeycomb cores. The honeycomb core performed better than the metallic foam core.	[54]
Sandwich panels with carbon/epoxy skins and an aluminium honeycomb core	Impact Loading (perforation—simulation)	Most of the impact energy was absorbed by the skins (between 80–90%)	[55]
Sandwich panels made from aluminum face sheets and honeycomb cores. Some cores reinforced with aluminium tubes.	Compression Impact testing—drop weight (crashworthiness—experiment)	The tube structure in some of the honeycomb cores exhibited higher and uniform energy absorption. Such structures had better impact resistance as well.	[56]
Sandwich panels consist of carbon and glass fiber face sheets and Nomex honeycomb core	Impact testing (perforation—experimental and simulation)	The honeycomb geometry (cell design) and core thickness effects the peak force and energy absorption capabilities of the sandwich panels.	[57]
Sandwich panels for both honeycomb core and face sheets made up from aluminium	Pendulum impact system (perforation—experimental and simulation)	Most of the impact energy was absorbed through plastic deformation by the face sheets and the core through plastic deformation. Different impact energies demonstrated different failure mechanisms.	[58]
Fiber metal laminates were used as skin on polypropylene honeycomb core to form a sandwich structure	Impact testing (perforation- experiment)	At low impact energies, there were only indentations on the front face sheet. Beyond the impact threshold energy, there was delamination of the skins and global bending of the structure.	[59]
Face sheets and honeycomb core for the sandwich panels made up from aluminum	Impact testing (perforation—experiment and simulation)	When the face sheet thickness were increased, it was found that the peak forces, SEA and EA also increases. Increasing the honeycomb cell size increases the SEA. The findings indicate that such sandwich panels can be optimized in terms of its design parameters to achieve excellent impact resistance.	[60]
Face sheets and honeycomb core for the sandwich panels made up from aluminum	Impact testing (perforation—experiment and simulation)	The structural integrity and stability was increased by reducing the cell size of the honeycomb. The height of the core does not affect the impact response or energy absorption.	[61]
Face sheets and honeycomb core for the sandwich panels made up from aluminum	High velocity Impact testing (perforation—experiment and simulation)	By increasing face sheet thickness and reducing honeycomb cell size, enhancement of perforation resistance of sandwich panels was achieved. The face sheets contributed most to energy absorption. Optimization of the face sheets and honeycomb design parameters are required to achieved the desired impact resistant.	[62]
Aluminum honeycomb sandwich structures with carbon fiber composite face sheets	Impact testing (perforation—experiment and simulation)	Impact response and damage behavior are affected by structural parameters. Face sheet thickness affects the impact resistance performance whereas honeycomb cell design has influence on the impact load.	[63]
The sandwich face sheet is from aluminum whereas the core is hybrid (corrugated thin aluminum plate and trapezoidal aluminium honeycomb	Compression Test (Crashworthiness—experiment and simulation)	The proposed system of honeycomb–corrugation hybrid structures are promising candidate energy absorbing applications. This is due to the complex deformation mechanism, which prevented honeycomb cell wall buckling.	[64]
The sandwich hybrid core is total aluminium consist of honeycomb and a grid of flat plates. The face sheets are from carbon fiber composites	Compression Test (Crashworthiness—experiment and simulation)	The combination of honeycomb and flat plats prevented both interfacial debonding and local buckling of core. This resulted in higher energy absorption.	[65]
Sandwich face sheets and honeycomb core made from aluminium filled with polyurethane foam	Impact testing (perforation—simulation)	The study found that the filling of honeycomb structure with high-density foam material had better energy absorption and impact resistance capabilities.	[66]
Sandwich panel made from aluminum (face sheets and core). The core was designed to be wavy.	Compression Test (Crashworthiness—simulation)	The sandwich panel had superior energy absorption capability compared with the conventional honeycomb sandwich panel, with a larger wave number and amplitude shows higher SEA.	[67]

**Table 4:** Sandwich structure with truss/lattice like structure as core.

Sandwich Construction	Loading/ Application	Summary of Findings	Reference
Aluminium pyramidal lattice core sandwich panel with polyurethane foam. Face sheets from carbon fiber composites	Impact testing (perforation—experiment and simulation)	The higher density of the pyramidal lattice structure exhibited better energy absorption capabilities	[68]
Aluminum sandwich panel composed of identical face sheets and tetrahedral lattice cores.	Air blast testing—ballistic pendulum system(Blast—experiment)	Tetrahedral lattice sandwich panels have better impulsive resistance than honeycomb structure made from the same material.	[69]
Sandwich panel consists of tetrahedral truss core and face sheet made of carbon/epoxy prepregs. Some of the samples were filled with polymer foam	Impact testing—gas gun (perforation—experiment)	The foam filled lattice structure had better impact resistance compared to the truss core structure only.	[70]
Sandwich panel consists of pyramidal lattice truss core and face sheet made of stainless steel. The voids are filled with ceramics and polymers	Impact testing (perforation—experiment)	Empty lattice structure does not contribute perforation. The filling of the voids with ceramic and polymers improves the impact resistance.	[71]
Sandwich structures with Y-shaped cores were fabricated using unidirectional carbon/epoxy prepreg	Compression Test (Blast/Crashworthiness—experimental and simulation)	Sandwich structures which had higher relative densities (more plies of fiber composites) had better energy absorption. However, no progressive collapse was observed.	[72]
Sandwich panel consists of aluminum pyramidal lattice truss core and face sheet made of carbon fiber composite	Compression test and low velocity impact (perforation/crashworthiness—experiment and simulation)	Under compression testing and impact test, the core structure failed in buckling. The higher the density of the core, the better the energy absorption.	[73]
Sandwich panel consists of hourglass and pyramidal lattice truss core and face sheet made of stainless steel	Underwater blast test (Blast—experiment)	The impact performance of the hourglass lattice panels was better than the pyramidal lattice panels.	[74]
Sandwich panel consists of pyramidal lattice truss core and face sheet made of stainless steel	Blast loading (Blast/perforation—experiment and simulation)	The impact resistance performance of the sandwich structure was found to be very similar to monolithic plates.	[75]
Lattice core sandwich cylinder made from aluminum both the core and inner/outer shell of cylinder	Internal Blast Loading (Blast—simulation)	The core geometry and core arrangement have significant effects on the blast resistance. The ticker the core wall, the less energy is absorbed. Asymmetrical design of the cylinder shells (inner and outer) enhances the blast resistance.	[76]
Sandwich panel consists of stainless steel pyramidal lattice structure as core and face sheet made of stainless steel	Underwater blast loading (Blast—experiment/simulation)	The front face sheet thickness influences the lattice structures’ deflection and energy absorption capabilities. An optimal value is desired for improving the impact resistance of the sandwich structure	[77]
Sandwich panels with lattice truss core filled by shear thickening fluid	Compressive Test (Blast—theoretical/simulation)	Energy absorption of panels increases with the increase of the fluid viscosity.	[78]
Sandwich panels made from Nylon with re-entrant Auxetic structure, the octet-truss structure and the BCC lattice structure for the core design.	Drop weight Compressive Test (Crashworthiness—experiment)	The geometrical design of the core structures significantly influences the impact energy absorption capabilities. The auxetic structure had better overall performance.	[79]

**Table 5:** Sandwich structure with origami/foldcore.

Sandwich Construction	Loading/ Application	Summary of Findings	Reference
Sandwich panels consists of aluminum origami core and aluminum face sheets	Impact testing gas gun (Perforation—experiment/simulations)	Deformation is localized without global deflection. Energy absorption was related to plastic deformation which dependent on the shape of the projectile. Perforation energy is linearly related to the origami wall thickness.	[80]
Aluminum Ron Resch origami core sandwich structure	Compression test (Crashworthiness—experiment/simulation)	The origami structure showed reduction in peak load due to formation of multiple plastic hinge lines. Overall, the performance of the origami panel is similar to the honeycomb structure	[81]
Sandwich panels of PET and PEEK foldcores with aluminum face sheets	Compression test (Crashworthiness—experiment/simulation)	It was found that the PEEK foldcores have better energy absorption capabilities. The geometrical parameters are crucial in developing and optimal design for energy absorption.	[82]
High Strength Low Alloy steel was used for the sandwich panel consisting of origami core and face sheets	Impulsive load test (Blast—simulation)	The panels consisting or the origami core are potential candidates for blast mitigation applications. The core pattern and geometry parameter influences the dynamic response of the panel.	[83]
High Strength Low Alloy steel was used for the sandwich panel consisting of origami core and face sheets	Compression and blast test (Blast/Crashworthiness—simulation)	For low to moderate load intensities, origami core absorbed more plastic energy than corresponding honeycomb core. The origami pattern and geometry parameters can be tailored to meet structural response requirements.	[84]
Sandwich panels consist of carbon fiber face sheets and foldcores made up from aramid paper	Impact test (Blast—experiment/simulation)	The foldcores core degradation and friction affects the sandwich’s energy absorption capabilities.	[85]
The sandwich core has a origami inspired honeycomb structure made from resins. Whereas the face sheets are made from aluminium alloy.	Drop-weight impact (Perforation—simulation)	The origami sandwich panel has better energy absorption characteristics than traditional honeycomb structure	[86]
Sandwich structure with foldcore/origami fabricated from brass H62.	Compression test (Crashworthiness–experiment/simulation)	The new *kirigami* inspired foldcore had better energy absorption characteristics than traditional honeycomb structure and *Miura-ori* core structure.	[87]
Sandwich beam with Miura-origami core and and face sheets made from steel	Blast loading (Blast—experiment)	The origami pattern (unit cell) has a wide range of parameters, which gives unique mechanical properties. This in returns affects the level of energy absorption.	[88]

**Table 6:** Sandwich structure with auxetic core.

Sandwich Construction	Loading/ Application	Summary of Findings	Reference
Sandwich panel with auxetic core structure, fabricated from PLA polymers (3D printed)	Impact Test (perforation—experiment/simulation)	The auxetic sandwich panel has good energy absorption capabilities	[89]
Sandwich panel with auxetic core structure, fabricated from aluminum	Impact Test (perforation–simulation)	The geometry parameters such as thicknesses and core density, affects the ballistic resistance performance. It was also found that auxetic honeycomb core as sandwich are good for ballistic protection.	[90]
Sandwich panel with auxetic core structure, fabricated from aluminum and face sheets from steel	Blast Loading (Blast—simulation)	When compared to monolithic plates, the auxetic sandwich panels absorb double the amount of impulsive energy via plastic deformation and significantly reduces the back facet’s maximum velocity.	[91,92,93]
3D printed polymeric PLA meta-sandwich structures made of cubic, octet and Isomax cellular cores	Impact test (perforation—experiment/simulation)	The core design geometrical parameters have significant effects on failure mechanism and energy absorption of the auxetic structures. Isomax cellular cores had the highest energy absorption capabilities.	[94]
Sandwich panels made from Nylon with re-entrant Auxetic structure, the octet-truss structure and the BCC lattice structure for the core design.	Drop weight Compressive Test (Crashworthiness—experiment)	The geometrical design of the core structures significantly influences the impact energy absorption capabilities. The auxetic structure had better overall performance.	[79]
Face sheets from braided fiber composites and sandwich aluminum core from auxetic 3D re-entrant lattices	Impact test (perforation—experiment/simulation)	Auxetic lattice core sandwich structure showed significant improvement in dissipating energy. The proposed sandwich design can be used for bulletproof body armors and safety vehicle parts.	[95]
Sandwich structure with auxetic, truss and hexagonal cores fabricated from polymer digital material and face sheets from carbon fiber composite.	Impact test (perforation—experiment)	The auxetic panel had lower impact resistance compared to the truss and hexagonal core panels. However, the auxetic panels had robustness and durability, because this panel was able to absorb multi hits	[96]
Sandwich panels made up from aluminum (face sheets and core). The core consists of a honeycomb with the re-entrant hexagonal cells.	Impact test (perforation—experiment/simulation)	The auxetic panels perform better than conventional honeycomb panels of the same size, areal density and material (in terms of blast resistance)	[97]
Sandwich panels with aluminium re-entrant hexagon honeycomb as core and steel face sheet	Impact blast (Blast—experiment/simulation)	The blast resistance performance indicators were more sensitive to thickness parameters.	[98]
Sandwich panels with auxetic chiral cellular structure core and face sheets were 3D printed from titanium alloy	Blast loading (Blast—experiment/simulation)	When thicker face sheet is used, the overall deformation was reduced, however highest SEA was achieved with thinner face plates. Chiral unit cell amplitudes affects the energy absorbing capabilities.	[99]
The entire sandwich panel (face sheets and auxetic core) was made from aluminum alloy	Blast loading (Blast—experiment/simulation)	The auxetic graded honeycomb cores with higher density on the upper layer had better blast resistance performance.	[100]
Sandwich panels with double arrowhead honeycomb. The core and the face sheets made from stainless steel.	Blast loading (Blast—experiment/simulation)	The blast resistance of this panel was enhanced by filling with polyurethane foam.	[101]
Sandwich panels fabricatedwith carbon/fiber epoxy composite face sheets, polyurethane rigid foam core or 3D printed PLAplastic cellular honeycombs head (hexagonal, re-entrant, hexachiral and arrowhead).	Impact test (perforation—experiment/simulation)	The arrowhead and hexachiral configurations are good for blast resistance applications involving impacts under large deformations.	[102]
The sandwich panel, including core (double-V auxetic structure cor) and face sheets, fabricated from a high-ductility stainless steel alloy.	Air blast loading (Blast—experiment/simulation)	The sandwich panel with auxetic core performed much better both in lightweight and protection than solid plate	[103]

**Table 7:** Sandwich structure with tubular core.

Sandwich Construction	Loading/ Application	Summary of Findings	Reference
Typical thin-walled square lattice truss tubes formed in a tubular sandwich manner	Compression test (Crashworthiness—simulation)	The sandwich wall has bending rigidity which improves the EA through shortening the wave length and improving the plastic bending moment	[104]
Sandwich structure comprise of tubes as core and thin face sheets riveted to the tubes. Material used is aluminium	Blast loading (Blast—simulation)	The effects of the spacing between the tubes affects the plastic hinges formation and interaction between the tubes.	[105]
Tubular composite structures made from carbon fiber composite with/without Nomex honeycomb sandwich core.	Impact Testing (Perforation—experiment)	The core material in the sandwich tubes absorbs more impact energy and resist severe local damage formation.	[106]
Aluminum foam sandwich tubes	Compression test (Crashworthiness—experiment)	Better energy absorption characteristics can be obtained by the proper thickness of metal foam core	[107,108]
Sandwich panels made from aluminum face sheets and honeycomb cores. Some cores reinforced with aluminium tubes.	Compression testing—drop weight (crashworthiness—experiment)	The tube structure in some of the honeycomb cores exhibited higher and uniform energy absorption. Such structures had better impact resistance as well.	[56]
The tubes are made from carbon fiber composite. The inner tube is filled with composite sandwich panels made from carbon fiber and Nomex honeycomb.	Compression testing (crashworthiness—experiment/simulation)	The tubes filled with the composite sandwich panels had better crash parameter performance	[109]
The tubes are made from carbon fiber composite. These tubes are inserted into the center of the honeycomb. Four types of honeycomb material was used. The face sheet was made from carbon fiber.	Compression testing (crashworthiness—experiment/simulation)	The tubes which were inserted into the center of the honeycomb structure had much better energy absorption capabilities.	[110]

**Table 8:** Sandwich structure with corrugated core.

Sandwich Construction	Loading/ Application	Summary of Findings	Reference
Kirigami modified corrugated core. Bothe the core and sandwich plates are made from aluminium.	Impact Perforation and Compression test by pendulum (Blast—experiment and simulation)	The sandwich made from Kirigami corrugated core had superior impact resistance when compared to panels with simple corrugated core.	[111]
3D printed sandwich panels where the core is bi-directional corrugated design. The material used was polycarbonate.	Compression test (Impact, Crashworthiness—experiment and simulation)	The sandwich structure with bi-directional corrugated core is obviously superior to the conventional sandwich structure with single-directional corrugated core.	[112]
Entire sandwich panels made from steel. The core is trapezoidal corrugation.	Impact Testing (Perforation—experiment and simulation)	The corrugated core design provided good shock absorption however; the performance was enhanced by filling with sand (dependent on density and stiffness of sand).	[113]
The sandwich panel (face sheets and corrugated core) was constructed from glass fiber reinforced polypropylene prepreg	Compression test (Crashworthiness—experimental and simulation)	Number of layered cores play an important role in the energy absorption capabilities. It was found that the way the corrugated cores are layered up affects the performance.	[114]
Curved sandwich panels—face sheets and axial/circular corrugated core made from carbon fiber composites.	Low velocity impact test (Blast/Impact—experiment/simulation)	The axially corrugated sandwich panels displayed good impact resistance characteristics.	[115]
E-glass sandwich panels for face sheets and corrugated cores: triangular, trapezoidal, rectangular were used. Some specimens had PVC foam filling.	Compression testing (crashworthiness—experiment)	Corrugated cores have higher energy absorption than just traditional foam filled sandwich structures. The number of unit cell/unit length and the corrugation angle plays a crucial role in the energy absorption performance. Plastic buckling of the corrugated cell walls are the initial failure modes observed.	[116]

## Data Availability

Not applicable.
